# LINC00941 Promotes Progression of Non-Small Cell Lung Cancer by Sponging miR-877-3p to Regulate VEGFA Expression

**DOI:** 10.3389/fonc.2021.650037

**Published:** 2021-03-31

**Authors:** Min-Huan Ren, Si Chen, Liang-Ge Wang, Wen-Xiu Rui, Pei Li

**Affiliations:** ^1^ Department of Respiratory Disease, Taikang Xianlin Drum Tower Hospital, Nanjing University School of Medicine, Nanjing, China; ^2^ Department of Infectious Diseases, Affiliated Hospital 2 of Nantong University, Nantong, China

**Keywords:** non-small cell lung cancer (NSCLC), LINC00941, angiogenesis, VEGFA, tumorigenesis

## Abstract

Long noncoding RNAs (lncRNAs) play critical roles in carcinoma occurrence and metastasis. LINC00941 has been found to mediate the development of gastric cancer, and LINC00941 was negatively associated with the longer overall survival of lung adenocarcinoma patients. Herein, our aim was to investigate the effects and mechanisms of LINC00941 in NSCLC progression. Microarray was used to identify the change lncRNAs in NSCLC, LINC00941 was found to increase in tumor tissues and patients’ plasma. Knockdown of LINC00941 didn’t modulate the proliferation of NSCLC cells, but inhibition of LINC00941 in NSCLC cells suppressed the angiogenesis ability of human umbilical vein endothelial cells (HUVECs). Moreover, LINC00941 promoted tumorigenesis *in vivo*, while si-LINC00941 inhibited tumor development of NSCLC. VEGFA was should to be significantly modulated by LINC00941 in NSCLC cells, then luciferase assay proved that LINC00941 regulated VEGFA expression *via* interacting with miR-877-3p. Followed functional experiments indicated that overexpression of LINC00941 accelerated angiogenesis and NSCLC tumor progression *via* miR-877-3p/VEGFA axis both *in vitro* and *in vivo*. In conclusion, our results clarified the LINC00941 function for the first time, and LINC00941 promoted the progression of NSCLC, which was mediated by miR-877-3p/VEGFA axis. This study might provide new understanding and targets for NSCLC diagnosis and treatment.

## Introduction

Lung cancer is the malignant tumor with the highest morbidity and mortality in the world, including non-small cell lung cancer (NSCLC) and small cell lung cancer, of which NSCLC accounts for about 85% (1). NSCLC diagnosis is often in the local late-stage or metastatic stage (2). Although the use of EGFR-TKIs has dramatically improved the clinical efficacy of NSCLC patients, those who are sensitive to treatment will inevitably acquire drug resistance after 10-16 months of treatment, resulting in disease progression five-year survival rate is only 15.9% (3). Therefore, the early diagnosis of recurrence and metastasis of lung cancer and the target mechanism of drug resistance treatment is an urgent problem to be solved.

Long non-coding RNA (lncRNA) can regulate multiple signal pathways and affect the occurrence and development of NSCLC. Pan et al. found that the expression of lncRNA NEAT1 in NSCLC tissues was significantly higher than that in paracancerous tissues, and it was positively correlated with age, vascular invasion, lymph node metastasis, and (TNM) stage of tumor lymph node metastasis (4). The level of circulating NEAT1 in plasma of patients with NSCLC was significantly increased, and the level of A549 cell line was also relatively high (4). Wang et al. found that MEG3 increased the sensitivity of NSCLC to cisplatin by regulating miR-21-5p/SOX7 pathway (5). The expression of MEG3 is directly regulated by miR-21-5p, miR-21-5p inhibits the effect of MEG3 on cisplatin resistance, SOX7 is the downstream target of miR-21-5p, and MEG3 regulates the expression of SOX7 by inhibiting miR-21-5p (5).

It has been confirmed that tumor angiogenesis plays a key role in the process of tumor invasion and metastasis (6). Tumor blood vessels not only promote tumor growth and invasion by transporting nutrients and metabolites but also provide channels for tumor cell metastasis. Moreover, the formation and growth of metastatic foci also depend on the formation of tumor blood vessels (7). Compared with other solid tumors, the microvessels in lung cancer tissues are more abundant and more prone to invasion and metastasis (8). Therefore, studying the mechanism of angiogenesis in lung cancer and taking corresponding interventions contribute to inhibit the angiogenesis of lung cancer, reduce the invasion and metastasis of lung cancer, and reduce the mortality of lung cancer. Vascular endothelial growth factor (VEGF) is a highly specific vascular endothelial mitogen, which is the most specific and powerful angiogenesis regulatory factor known at present (9). Studies have shown that the expression of VEGF subtypes plays an important role in the occurrence and development of tumors (10–12). After binding to its specific receptor VEGFR, VEGF induces vascular endothelial cell division and proliferation, promotes endothelial cell metastasis, and enhances capillary permeability and plasma exudation through a series of regulatory mechanisms and biofeedback, thus regulating tumor formation, invasion, and metastasis (13).

LINC00941 has been found to mediate the development of gastric cancer (14), head and neck squamous cell carcinoma (15), and papillary thyroid cancer (16). Moreover, LINC00941 was negatively associated with the longer overall survival of lung adenocarcinoma patients (17). However, the underlying mechanism of LINC00941 in NSCLC remains unclear. In the present study, we found LINC00941 was highly expressed in NSCLC tissues. And LINC00941 promoted the progression of NSCLC *via* modulating angiogenesis, which might provide potential biomarkers and novel insight for NSCLC treatment.

## Materials and Methods

### Patients Experiments

The para-carcinoma tissues and cancer tissue of 40 NSCLC patients were collected, and plasma of 40 NSCLC patients and 20 volunteers were isolates, which were used for follow-up experimental detection. The experiment was permitted by the Ethics Review Committee of Taikang Xianlin Drum Tower Hospital, Nanjing University School of Medicine and the patients signed informed consent.

### Cell Culture and Transfection

The commonly used A549 and NCI-1299 cell lines of NSCLC and human umbilical vein endothelial cells (HUVECs) were purchased from the ATCC cell center of The United States. The cells were cultured in RPMI 1640 medium with 10% fetal bovine serum and 1% dual-antibody solution in an incubator at 37°C and 5% CO2. The culture medium was changed every day and passed every 3 days. 500 nM miRNA or or 2 μg siRNA was transfected into cells, which was mediated by LipofectamineTM 2000 (Invitrogen, Carlsbad, CA, USA). And plasmid or miRNA or small interfering RNA (si-RNA) were constructed and purchased from by Ribobio company (Guangzhou, China).

### Animals Experiments

A549/NCI-1299 cells of each group were prepared for inoculation for subculture for 15 generations, and the concentration was adjusted to 5x10^7^/0.1ml/site and then divided into different packs. The cell suspension is blown away. Eighteen BALB/C female nude mice 4-5 weeks, weight around 20g) were selected and grouped and numbered. Each nude mouse was weighed. The right armpit was disinfected with 75% alcohol, and 0.1ml cell suspension was injected. After 7 days, mice were treated intraperitoneally with bevacizumab (10 mg/kg), and agomiR-877-3p (10 μmol) was injected through tail vein. Then, the mice were observed daily. After 4 weeks, the nude mice were collected and killed by excessive carbon dioxide, the tumors were removed, and the tumors were photographed, weighed and recorded after all the surrounding connective tissue was removed. Present study was approved by Institutional Animal Care and Use Committee of Taikang Xianlin Drum Tower Hospital, Nanjing University School of Medicine.

### qRT-PCR

We used trizol method to extract RNA in tissues and cells, and RNA concentration and purity were determined using NanoDrop 2000 (Thermo Scientific, USA). RNA is used as transcription template to reverse transcribe into cDNA. Then SYBR Premix Ex TaqII was selected for RT PCR reaction. The expression value of the normal group was set as 1, and the relative expression of the experimental group was expressed as 2^-△△CT^. GAPDH was used as internal control.

### *In Situ* Hybrization (ISH) Assay

ISH assay used to identify the location of LINC00941 in lung tissues (18). Lung sections were treated with 0.05% H_2_O_2_, followed by digestion with pepsin at 37°C for 1–2 min, and then terminated and washed with 0.5 M PBS for 15 min. Next, the sections were incubated in prehybridization buffer for 2 h at 37°C and in hybridization buffer overnight at 37°C. The sections were washed thoroughly with a gradient of SSC to remove the background signals. Next, the sections were treated with a biotinylated digoxin antibody at 37°C for 2 h. After the sections were thoroughly washed with PBS, the samples were incubated with streptavidin-biotin complex for 30 min and biotinylated peroxidase for 30 min at 37°C. The color reaction was developed using the DAB substrate, and the sections were dehydrated and then mounted with neutral gum.

### MTT Assay

MTT assay used to determine the proliferative ability of cells. 100 μL (1×10^4^ cells) were inoculated in 96-well plates and cultured at 37°C with 5% CO_2_ for 24 h. At 24h after transfection, 50 μL MTT solution (5 mg/mL) was added to each well, and the supernatant was discarded after incubation for 4h at 37°C. The reduction reaction was terminated by adding 150 μL dimethyl sulfoxide (DMSO) to each well. The 96-well plate was continuously shaken for 30 min, and the optical density value of each well at 570 nm wavelength was determined by ELISA, and the average value of each group was taken.

### Matrix Assay

Matrix assay used to determine the tube formation ability of HUVECs. The GFR BD Matrigel was placed in a refrigerator at 4° for 24 h and thawed into a gelatinous liquid. The gelatin-like liquid was rapidly added to the 4° precooled porous plate with 200 μL/well, and the plate was horizontally oscillated. After the plate was leveled, the Matrigel was placed into a 37° cell incubator for 45 min and the Matrigel was solidified. HUVEC cells in each group were fused to reach about 80%, digested with 0.25% pancreatic enzyme +EDTA, washed with PBS solution for 3 times, resuspended with the supernatant of NSCLC cells, counted cells under inverted microscopic field, and adjusted cell density to 1×10^4^/mL. The cell suspension of each group was transferred into the culture plate and each well was 3 ml. Place the culture plate into the cell incubator for culture. After 24 h, lumen formation was observed under an inverted microscope at low magnification and photographed under magnification.

### Transwell Assay

The matrix glue and basic medium 1640 were fully mixed according to 1:3. A mixture of 50 μL matrix glue and basal medium 1640 was added to the bottom of the chamber. The culture plates with small Chambers were placed in a 5% CO_2_ incubator for 30 minutes. Single-cell suspension was prepared and the cell concentration was adjusted to lx10^5^/mL. In the 24-well plate, a small chamber with and without coated matrix glue was set, and a complete medium containing 10% serum was added. Cell suspension of 200 μL was slowly added into a small chamber and cultured at 37°C and 5% CO_2_ for 24 h. The cells in the small chamber were removed with a wet cotton swab and fixed immediately with formaldehyde for 5 minutes. After that, the small chamber was taken out and dried. Crystal violet was used for dyeing for 20 min. Then, the chamber was rinsed with water and dried. The number of transmembrane cells was observed and counted under the microscope.

### Luciferase Assay

Luciferase assay used to test the binding between miR-877-3p and LINC00941/VEGFA, HEK293 cells were co-transfected with 20 mmol/L miR-877-3p mimic or miR-NC together with WT-LINC00941/Mut-LINC00941 or WT-VEGFA/Mut-VEGFA. Luciferase activity was measured with Dual Luciferase Reporter Assay Kit (Transgene, China) on GloMax20/20 at 48 h after the transfection.

### Immunohistochemical (IHC) Staining

Paraffin sections of carcinoma were dewaxing to water in xylene and descending series of ethanol. We penetrated sections using 0.5% Triton X-100. After 3 times wash, we blocked sections with 50% goat serum. Then, sections were incubated with Ki67 antibody (ab15580, Abcam) or VEGFA antibody (ab1316, Abcam) overnight. The second day, tumor sections were incubated with secondary antibody for 1h, then stained nuclei using DAPI for 5 min. Then, IHC staining positive region was detected by microscope under light scope.

## Statistical Analysis

Data were shown as mean ± SD. Student’s t-test or one-way ANOVA was used to compare the groups. P<0.05 was considered significance. All experiments were repeated three times.

## Results

### LINC00941 Was Increased in NSCLC Tissues

**Figure 1:**
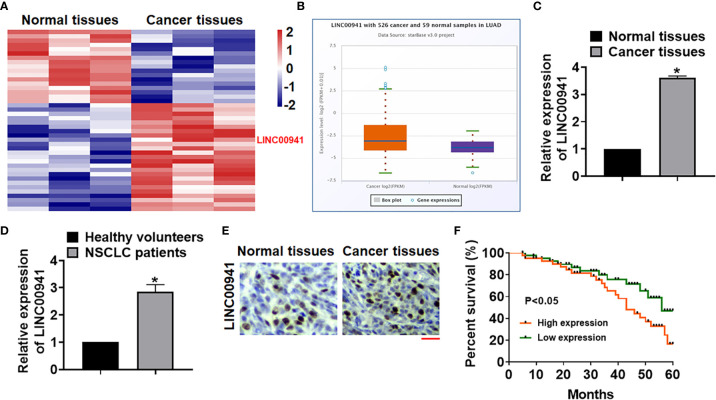
The expression of LINC00941 in NSCLC tissues. **(A)** LncRNA expression profiles in normal and cancer tissues of NSCLC patients. **(B)** ENCORI website showed LINC00941 level in cancer and normal samples of lung adenocarcinoma (LUAD). **(C)** The expression of LINC00941 in normal and cancer tissues was detected by qRT-PCR. **(D)** RNA in plasma of healthy volunteers and NSCLC patients was isolated, and LINC00941 expression was calculated. **(E)** ISH analysis for LINC00941 expression normal and cancer tissues of NSCLC patients. Scale bar, 200 μm. **(F)** 5-years’ survival rate of NSCLC patients with high and low expression of LINC00941. Data are mean ± SD; *P < 0.05. All experiments were repeated three times.

Bioinformatic analysis was performed to identify the differentially expressed lncRNAs in normal and cancer tissues of NSCLC, which showed an increase of LINC00941 in NSCLC tissues (**Figure 1A**). ENCORI website showed that LINC00941 level was significantly increased in cancer tissues comparing with normal samples of lung adenocarcinoma (LUAD) (**Figure 1B**). As well, the para-cancer tissues and cancerous tissues of NSCLC patients in our hospital were collected, and qRT-PCR also indicated that LINC00941 was upregulated in cancer tissues of NSCLC patients (**Figure 1C**). Then, plasma of NSCLC patients and healthy volunteers was collected, and qRT-PCR data suggested that LINC00941 was high expressed in plasma of NSCLC patients comparing with healthy volunteers (**Figure 1D**). ISH assay used to identify the location and expression of LINC00941 in NSCLC tissues, which showed that LINC00941 mainly located in cytoplasm and was significantly increased in cancer tissues (**Figure 1E**). Survival analysis was used for high and low expression of LINC00941 in LUAD, and data showed that patients with high level of LINC00941 had a lower survival rate (**Figure 1F**).

### LINC00941 Promoted Angiogenesis of Vascular Endothelial Cells

**Figure 2:**
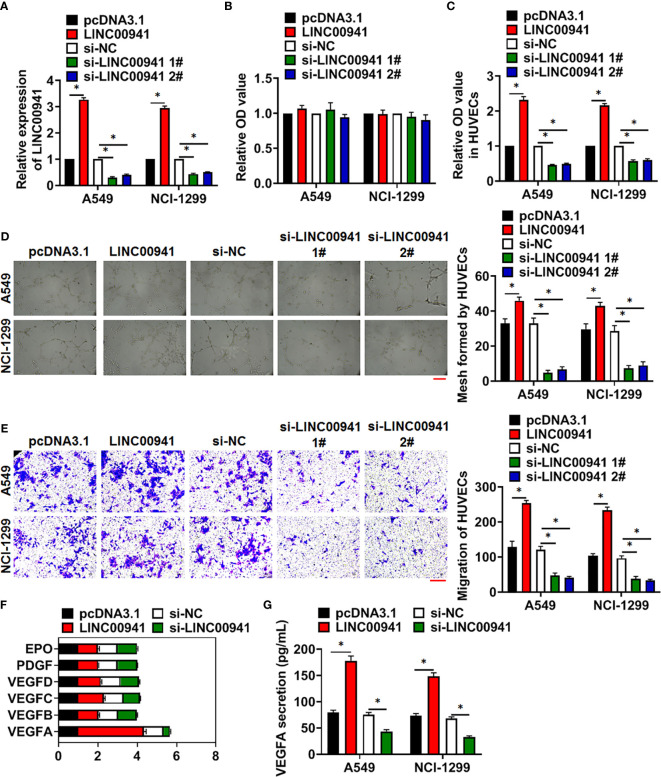
LINC00941 in NSCLC cells promoted angiogenesis of HUVECs. Stable LINC00941 knockdown A549 and NCI-1299 cells, and exogenously overexpressed LINC00941 in A549 and NCI-1299 cells were constructed. **(A)** The expression of LINC00941 was determined by qRT-PCR. **(B)** MTT assay used to detect proliferation of A549 and NCI-1299 cells. HUVECs were cultured with supernatant of A549 and NCI-1299 cells stably expressed LINC00941 or si-LINC00941. **(C)** MTT assay used to detect proliferation of HUVECs. **(D)** Effect of LINC00941 expression in NSCLC cells on HUVECs in tube formation assay. Scale bar, 100 μm. **(E)** Transwell assay was to examine invasion of HUVECs. Scale bar, 100 μm. **(F)** The expression of the proliferation and migration-related genes in HUVECs. **(G)** The level of VEFGA in NSCLC cells in ELISA assay. Data are mean ± SD; *P < 0.05. All experiments were repeated three times.

To identify the effect of LINC00941, we constructed stable LINC00941 knockdown A549 and NCI-1299 NSCLC cells endogenously silencing LINC00941, and exogenously overexpressed LINC00941 in A549 and NCI-1299 cells *via* retroviral infection, qRT-PCR showed the LINC00941 level in cell lines (**Figure 2A**). Followed assay showed that overexpression or knockdown of LINC00941 had a little effect on cell proliferation (**Figure 2B**). Considering the essential role of angiogenesis in cancer development (19), we cultured human umbilical vein endothelial cells (HUVECs) with supernatant of A549 and NCI-1299 cells with LINC00941 or si-LINC00941. MTT assay showed that LINC00941 in NSCLC cells promoted the proliferation of HUVECs, while si-LINC00941 in NSCLC cells inhibited the proliferation of HUVECs (**Figure 2C**). Tube formation assay revealed that overexpression of LINC00941 promoted mesh formation, and transwell assay indicated that LINC00941 enhanced the migration ability of HUVECs, while silencing LINC00941 yielded the opposite effects (**Figures 2D, E**). To explore the underlying mechanism, we analyzed several proliferation and migration-related genes in vascular endothelial cells, VEGFA was obviously correlated with the variation in LINC00941 expression (**Figure 2E**). ELISA also indicated that the VEGFA level was dramatically modulated by the LINC00941 (**Figures 2F, G**).

### Inhibition of LINC00941 Suppressed Tumorigenesis of Lung Cancer

**Figure 3:**
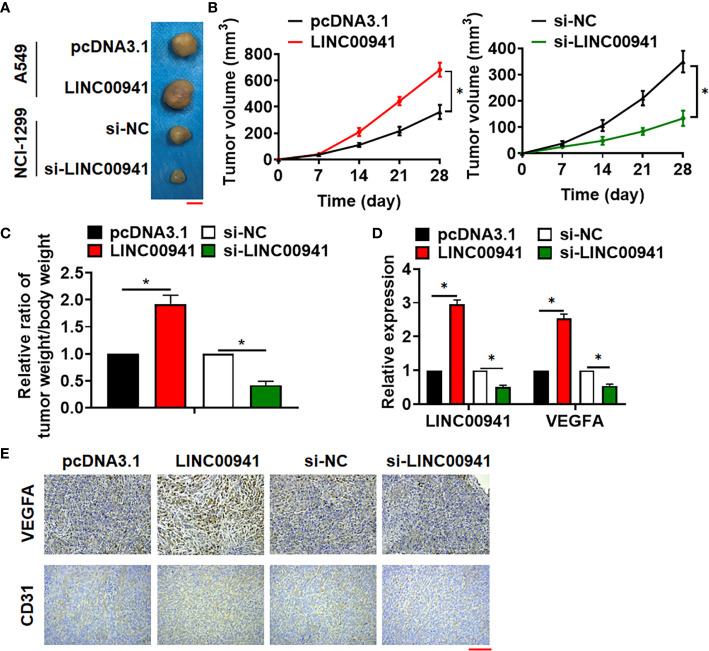
Silencing of LINC00941 inhibited tumorigenesis of NSCLC. **(A)** Images of the representative excised tumors from the mice at 28 days after injection with the A549 (stably expressed LINC00941) and NCI-1299 cells (stably expressed si-LINC00941). **(B)** Tumor volume was measured every 7 days. **(C)** The ratio of tumor weigh to body weight was calculated. **(D)** The mRNA of LINC00941 and VEGFA in isolated tumors were detected by qRT-PCR. **(E)** IHC staining used to detect VEGFA and CD31 expression of isolated tumors. Data are mean ± SD; *P < 0.05. All experiments were repeated three times.

Tumor formation in nude mice was performed to evaluate LINC00941 function *in vivo*. A549 cells with LINC00941 and NCI-1299 cells with si-LINC00941 were subcutaneously injected into right lower limb of the nude mice. 4 weeks later, tumors were isolated (**Figure 3A**). LINC00941 enhanced tumor volume, and increased the ratio of tumor weight to body weight, while si-LINC00941 exerted the opposite effects (**Figures 3B, C**). In addition, LINC00941 induced LINC00941 expression in isolated tumor tissues, while si-LINC00941 reduced LINC00941 level after injection (**Figure 3D**). Moreover, IHC data indicated that LINC00941 promoted VEGFA expression in tumors, and si-LINC00941 inhibited VEGFA level (**Figure 3E**). And LINC00941 promoted CD31 expression in tumors, which indicated a higher ability of vascularization (**Figure 3E**).

### LINC00941 Regulated VEGFA Expression *via* Sponging miR-877-3p

**Figure 4:**
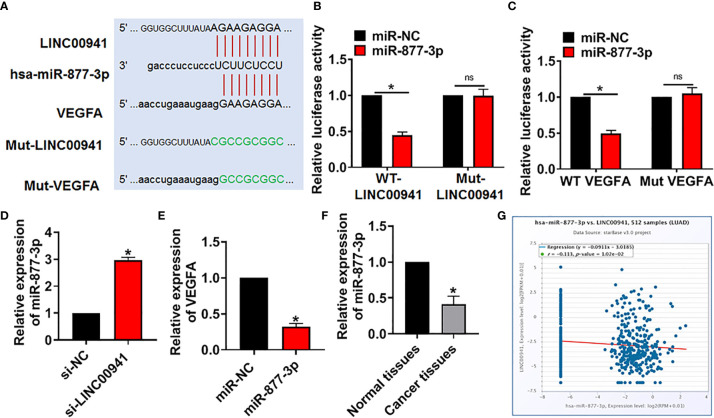
LINC00941 acted as a competitive endogenous RNA for miR-877-3p and modulated VEGFA level. **(A)** Bioinformatics used to predict the binding bases of miR-877-3p and LINC00941/VEGFA. **(B)** Wild type and mutant LINC00941 was transfected into HEK293 cells with or without miR-877-3p, and luciferase assay was to evaluate the binding between miR-877-3p and LINC00941. **(C)** Luciferase assay was to test the binding between miR-877-3p and VEGFA. **(D)** A549 cells were transfected with si-LINC00941/si-NC, the expression of miR-877-3p was tested. **(E)** The expression of VEGFA was determined after miR-877-3p mimic transfection in A549 cells. **(F)** The level of miR-877-3p in normal and cancerous tissues of NSCLC patients was detected. **(G)** ENCORI website used to determine the correlation between LINC00941 and miR-877-3p in lung Adenocarcinoma (LUAD). Data are mean ± SD; *P < 0.05, ns, no statistical significance. All experiments were repeated three times.

At present, it has been found that lncRNA regulates miRNA by acting as sponge of miRNA (20). Through the prediction of the bioinformatics website (DIANA, Starbase, Targetscan), we found that miR-877-3p may be a target of LINC00941 in regulating VEGFA level (**Figure 4A**). The binding site was predicted and luciferase assay reported that miR-877-3p could inhibit the luciferase activity of WT-LINC00941, but not Mut-LINC00941 (**Figure 4B**). As well, miR-877-3p targeted and bound with VEGFA (**Figure 4C**). After transfection with si-LINC00941/si-NC in A549 cells, the expression of miR-877-3p was increased in the si-LINC00941 group (**Figure 4D**). The expression of VEGFA was decreased after miR-877-3p mimic transfection (**Figure 4E**). In NSCLC patient’s tissues, the level of miR-877-3p was significantly reduced (**Figure 4F**). Correlation analysis using ENCORI website showed that there was a negative correlation between LINC00941 and miR-877-3p in lung adenocarcinoma (LUAD) (**Figure 4G**). Taken together, LINC00941 could regulate VEGFA level *via* sponging miR-877-3p.

### LINC00941 Promoted Angiogenesis of Vascular Endothelial Cells *via* miR-877-3p/VEGFA Axis

**Figure 5:**
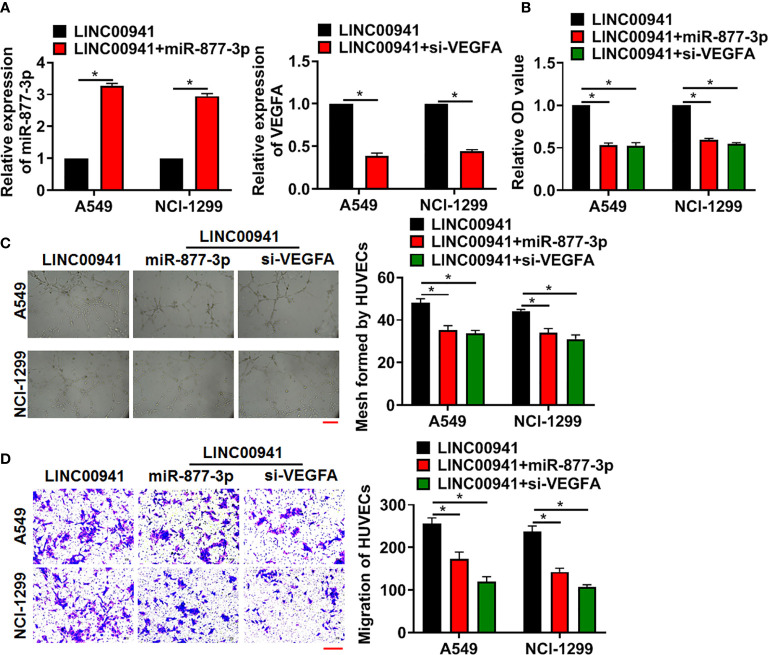
LINC00941 promoted angiogenesis through miR-877-3p/VEGFA axis. A549 and NCI-1299 cells (stably expressed LINC00941) were transfected with miR-877-3p or si-VEGFA. **(A)** The transfection efficiency of miR-877-3p or si-VEGFA was detected using qRT-PCR. HUVECs were cultured with supernatant of A549 and NCI-1299 after transfection. **(B)** MTT assay used to detect proliferation of HUVECs. **(C)** Tube formation assay was to test angiogenesis in HUVECs. Scale bar, 100 μm. **(D)** Transwell assay was to examine invasion of HUVECs. Scale bar, 100 μm. Data are mean ± SD; *P < 0.05. All experiments were repeated three times.

Next, we forced miR-877-3p or inhibited VEGFA level in A549 and NCI-1299 cells with LINC00941, the miR-877-3p mimics transfection increased miR-877-3p level, and si-VEGFA inhibited VEGFA level in A549 and NCI-1299 cells (**Figure 5A**). Then, HUVECs were cultured with supernatant of A549 and NCI-1299 cells after transfection. Followed functional experiments indicated that overexpression of miR-877-3p or inhibition of VEGFA suppressed the proliferation, mesh formation and migration of HUVECs (**Figures 5B–D**).

### LINC00941 Promoted Tumorigenesis of NSCLC *via* Modulating miR-877-3p/VEGFA

**Figure 6:**
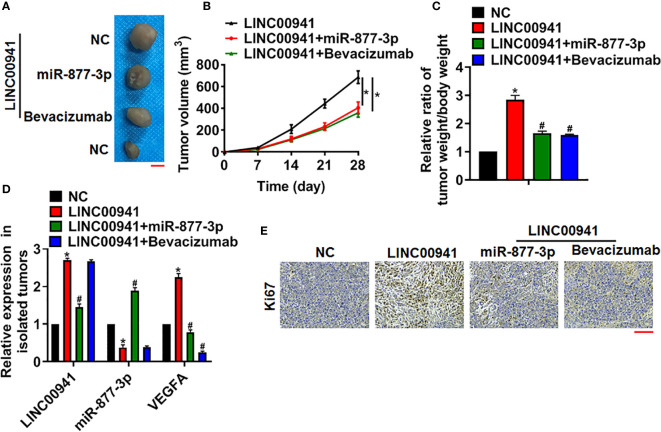
LINC00941 promoted NSCLC growth *in vivo via* miR-877-3p/VEGFA axis. A549 cells stably expressed LINC00941 were subcutaneously injected into right lower limb of the nude mice. 7 days later, agomiR-877-3p was injected through tail vein, or bevacizumab (10 mg/kg) was intraperitoneally injected. **(A)** Images of the representative excised tumors at 28 days after injection. **(B)** Tumor volume was measured every 7 days. **(C)** The ratio of tumor weigh to body weight was calculated. **(D)** The mRNA of LINC00941, miR-877-3p and VEGFA in isolated tumors were detected by qRT-PCR. **(E)** Ki67 staining used to detect the proliferation of isolated tumors. Data are mean ± SD; *P < 0.05 vs NC, ^#^P < 0.05 vs LINC00941. All experiments were repeated three times.

To investigate the pathophysiological function of LINC00941 on the tumorigenesis, A549 cells with LINC00941/NC were subcutaneously injected into right lower limb of the nude mice. 7 days later, agomiR-877-3p was injected through tail vein, or bevacizumab (VEGF monoclonal antibody) was intraperitoneally injected. 4 weeks later, tumors were isolated and showed in **Figure 6A**. Overexpression miR-877-3p or inhibition of VEGFA decreased tumor volume and tumor weight (**Figures 6B, C**). Then, the levels of LINC00941, miR-877-3p and VEGFA in isolated tumors were determined using qRT-PCR (**Figure 6D**). Moreover, Ki67 staining used to evaluate proliferation level of tumors. Data showed that LINC00941 promoted cancer cells proliferation, while agomiR-877-3p or si-VEGFA reversed the accelerating effects of LINC00941 in NSCLC progression (**Figure 6E**).

## Discussion

Lung cancer is the malignant tumor with the highest morbidity and mortality in the world (21). Although various treatment measures have made great progress, the 5-year survival rate after lung cancer operation is very low, and the treatment effect is not good. One of the main reasons for the low 5-year survival rate of patients with lung cancer is the metastasis of cancer cells (22). Therefore, the intervention of cancer cell metastasis is of great significance to improve the prognosis and reduce the mortality.

The metastasis of cancer is a complex process involving multiple factors, including proliferation and differentiation of tumor cells, neovascularization induced by tumor cells, matrix degradation by related proteases secreted by tumor cells, and so on (23). At present, it has been confirmed that tumor-related neovascularization plays an important role in the process of tumor invasion and metastasis (24). Neovascularization not only provides sufficient nutrition for the growth of primary or metastatic tumors, promotes the occurrence and development of tumors, but also provides channels for tumor metastasis (25). Angiogenesis is an indispensable factor in tumor growth and metastasis. And many studies have pointed out that tumor-related neovascularization is closely related to the growth, invasion, metastasis and prognosis of NSCLC (26, 27).

LncRNA, which contains more than 200bp, is not involved in coding proteins, but can regulate gene expression at all levels in the form of RNA (28). According to research, the biological process and abnormal expression of lncRNA are related to cancer and play an important role in the occurrence and development of tumors (29). LncRNA can affect a variety of signal pathways and thus participate in the occurrence and development of NSCLC (30). Based on the stability and specificity of lncRNA, lncRNA may become a diagnostic marker of NSCLC and improve the detection rate of NSCLC. And in present study, we first used microarray to identify the differentially expressed lncRNAs in NSCLC tissues, which showed a significant increase of LINC00941 in cancer tissues. Particular, LINC00941 could be found in plasma, and NSCLC patients had a high LINC00941 expression. We also found that LINC00941 was related to poor prognosis of NSCLC. Therefore, present study had some clinical relevance. However, due to the small patient sample of present study, we cannot draw conclusions about correlation with individual patient outcomes, but we now comment on this limitation and suggest future studies that could examine more details about LINC00941 with NSCLC patients.

LINC00941 was first found in lung adenocarcinoma (LUAD), and lung adenocarcinoma belongs to NSCLC (31). Subsequent studies indicated that LINC00941 was correlated with invasion and lymphatic metastasis of gastric cancer (14). Moreover, LINC00941 promoted the metastasis of papillary thyroid cancer by modulating CDH6 (16), and LINC00941 facilitated the metastasis of colorectal cancer through activating the TGF-β/SMAD2/3 pathway (32). These studies suggested that LINC00941 was related to cancer invasion and metastasis. However, the role and mechanism of LINC00941 in the metastasis of NSCLC has not been reported.

Numerous reports show that abnormal expression of genes in tumors indicates malignancy of tumor cells (33). In order to clary the effect of LINC00941 in the malignancy of NSCLC cells, we constructed stable LINC00941 knockdown A549 and NCI-1299 NSCLC cells, and exogenously overexpressed LINC00941 in A549 and NCI-1299 cells. This method is common for exploring genes function in tumor cell, and A549 and NCI-1299 are two usual NSCLC cell lines. Surprisingly, the data showed that overexpression or inhibition of LINC00941 in NSCLC cells had no effects on their proliferative ability. Considering the essential role of tumor-related neovascularization in the process of tumor invasion and metastasis, we cultured HUVECs with supernatant from NSCLC cells with overexpression or inhibition of LINC00941. Then, functional experiment showed that LINC00941 in NSCLC cells promoted the proliferation, tube formation and migration of HUVECs. As well, *in vivo* tumor formation experiments were also confirmed LINC00941 promoted oncogenesis in nude mice, while si-LINC00941 suppressed oncogenesis. These data indicated that LINC00941 in NSCLC cells might promoted tumor development through activating angiogenesis process.

In order to claim the mechanism of LINC00941 in angiogenesis, we analyzed several proliferation and migration-related genes in vascular endothelial cells, VEGFA was obviously correlated with the variation in LINC00941 expression. VEGFA is a member of vascular endothelial growth factors (VEGFs), and is the most specific, powerful and important angiogenic regulator (34). The biological effects of VEGFA and VEGFR are not exerted until they are combined with each other. VEGFR is not only expressed in lung cancer cells, but also expressed in a variety of other cells, including macrophages, monocytes (35). Shi W et al. used siRNA of VEGFA to treat gastric cancer and found that renal cancer cells cultured *in vitro* showed selective VEGFA, in the tumor model of nude mice, inhibiting tumor growth by reducing the expression of VEGFA in tumor cells (36). A study on colorectal cancer focused on the retrospective analysis of the relationship between VEGFA, its receptor and clinicopathology. The results showed that VEGFA was related to the degree of tumor differentiation (37). These results suggest that the expression level of VEGFA may be used as a biomarker of tumor malignant invasion and has prognostic value. In addition, studies have pointed out that lncRNA, like mRNA, can bind with miRNA to inhibit the down-regulation of mRNA expression by miRNA, thus realizing the regulatory function. Thus, we used bioinformatics website to analyze miRNAs that sponged by LINC00941 and targeted VEGFA. The data showed that miR-877-3p had complementary base pairs with LINC00941 and VEGFA. Luciferase assay proved that LINC00941 regulated VEGFA expression *via* interacting with miR-877-3p. Moreover, miR-877-3p was decreased in NSCLC tissues, and negatively associated with LINC00941. Followed functional experiments indicated that overexpression of LINC00941 accelerated angiogenesis and NSCLC progression *via* miR-877-3p/VEGFA axis both *in vitro* and *in vivo*.

## Conclusion

In conclusion, our results clarified the LINC00941 function for the first time, and LINC00941 promoted the progression of NSCLC, which was mediated by miR-877-3p/VEGFA axis. This study might provide new understanding and targets for NSCLC diagnosis and treatment.

## Data Availability Statement

The original contributions presented in the study are included in the article/supplementary material. Further inquiries can be directed to the corresponding author.

## Ethics Statement

The studies involving human participants were reviewed and approved by Taikang Xianlin Drum Tower Hospital, Nanjing University School of Medicine. The patients/participants provided their written informed consent to participate in this study. The animal study was reviewed and approved by Taikang Xianlin Drum Tower Hospital, Nanjing University School of Medicine.

## Author Contributions

M-HR and SC designed the study. L-XW performed experiments. W-XR collected and analyzed data. PL wrote the manuscript. All authors contributed to the article and approved the submitted version.

## Conflict of Interest

The authors declare that the research was conducted in the absence of any commercial or financial relationships that could be construed as a potential conflict of interest.

## References

[1] Topalian S Hodi F Brahmer J Gettinger S Smith D McDermott D. Safety, activity, and immune correlates of anti-PD-1 antibody in cancer. N Engl J Med (2012) 366(26):2443–54. 10.1056/NEJMoa1200690 PMC3544539 22658127

[2] Morgensztern D Ng S Gao F Govindan R. Trends in stage distribution for patients with non-small cell lung cancer: a National Cancer Database survey. J Thorac Oncol (2010) 5(1):29–33. 10.1097/JTO.0b013e3181c5920c 19952801

[3] Ettinger D Akerley W Borghaei H Chang A Cheney R Chirieac L. Non-small cell lung cancer, version 2.2013. J Natl Compr Cancer Network JNCCN (2013) 11(6):645–53; quiz 653. 10.6004/jnccn.2013.0084 23744864

[4] Pan L Zhong T Tang R Li P Dang Y Huang S. Upregulation and clinicopathological significance of long non-coding NEAT1 RNA in NSCLC tissues. Asian Pacific J Cancer Prev APJCP (2015) 16(7):2851–5. 10.7314/APJCP.2015.16.7.2851 25854373

[5] Wang P Chen D Ma H Li Y. LncRNA MEG3 enhances cisplatin sensitivity in non-small cell lung cancer by regulating miR-21-5p/SOX7 axis. OncoTargets Ther (2017) 10:5137–49. 10.2147/OTT.S146423 PMC5661845 29123412

[6] Dong H Weng C Bai R Sheng J Gao X Li L. The regulatory network of miR-141 in the inhibition of angiogenesis. Angiogenesis (2019) 22(2):251–62. 10.1007/s10456-018-9654-1 30465119

[7] Martial S. Involvement of ion channels and transporters in carcinoma angiogenesis and metastasis. Am J Physiol Cell Physiol (2016) 310(9):C710–27. 10.1152/ajpcell.00218.2015 26791487

[8] Yuan A Yang P Yu C Lee Y Yao Y Chen C. Tumor angiogenesis correlates with histologic type and metastasis in non-small-cell lung cancer. Am J Respir Crit Care Med (1995) 152:2157–62. 10.1164/ajrccm.152.6.8520790 8520790

[9] Cressey R Wattananupong O Lertprasertsuke N Vinitketkumnuen U. Alteration of protein expression pattern of vascular endothelial growth factor (VEGF) from soluble to cell-associated isoform during tumourigenesis. BMC cancer (2005) 5:128. 10.1186/1471-2407-5-128 16202150 PMC1262699

[10] Le X Nilsson M Goldman J Reck M Nakagawa K Kato T. Dual EGFR/VEGF pathway inhibition: a promising strategy for patients with EGFR-mutant NSCLC. J Thorac Oncol (2020) 16:205–15. 10.1016/j.jtho.2020.10.006 33096270

[11] Chen C Luo Y He W Zhao Y Kong Y Liu H. Exosomal long noncoding RNA LNMAT2 promotes lymphatic metastasis in bladder cancer. J Clin Invest (2020) 130(1):404–21. 10.1172/JCI130892 PMC6934220 31593555

[12] Zizza P Dinami R Porru M Cingolani C Salvati E Rizzo A. TRF2 positively regulates SULF2 expression increasing VEGF-A release and activity in tumor microenvironment. Nucleic Acids Res (2019) 47(7):3365–82. 10.1093/nar/gkz041 PMC6468246 30698737

[13] Spagnuolo A Palazzolo G Sementa C Gridelli C. Vascular endothelial growth factor receptor tyrosine kinase inhibitors for the treatment of advanced non-small cell lung cancer. Expert Opin Pharmacother (2020) 21(4):491–506. 10.1080/14656566.2020.1713092 31957503

[14] Luo C Tao Y Zhang Y Zhu Y Minyao D Haleem M. Regulatory network analysis of high expressed long non-coding RNA LINC00941 in gastric cancer. Gene (2018) 662:103–9. 10.1016/j.gene.2018.04.023 29653230

[15] Hu Y Guo G Li J Chen J Tan P. Screening key lncRNAs with diagnostic and prognostic value for head and neck squamous cell carcinoma based on machine learning and mRNA-lncRNA co-expression network analysis. Cancer Biomarkers Section A Dis Markers (2020) 27(2):195–206. 10.3233/CBM-190694 PMC12662289 31815689

[16] Gugnoni M Manicardi V Torricelli F Sauta E Bellazzi R Manzotti G. Linc00941 Is a Novel Transforming Growth Factor β Target That Primes Papillary Thyroid Cancer Metastatic Behavior by Regulating the Expression of Cadherin 6. Thyroid (2020) 31:247–63. 10.1089/thy.2020.0001 32495722

[17] Wang L Zhao H Xu Y Li J Deng C Deng Y. Systematic identification of lincRNA-based prognostic biomarkers by integrating lincRNA expression and copy number variation in lung adenocarcinoma. Int J Cancer (2019) 144(7):1723–34. 10.1002/ijc.31865 30226269

[18] Kalmár A Nagy Z Galamb O Csabai I Bodor A Wichmann B. Genome-wide expression profiling in colorectal cancer focusing on lncRNAs in the adenoma-carcinoma transition. BMC Cancer (2019) 19(1):1059. 10.1186/s12885-019-6180-5 31694571 PMC6836529

[19] Chen J Liu A Wang Z Wang B Chai X Lu W. LINC00173.v1 promotes angiogenesis and progression of lung squamous cell carcinoma by sponging miR-511-5p to regulate VEGFA expression. Mol Cancer (2020) 19(1):98. 10.1186/s12943-020-01217-2 32473645 PMC7260858

[20] Jin D Guo J Wu Y Du J Yang L Wang X. mA mRNA methylation initiated by METTL3 directly promotes YAP translation and increases YAP activity by regulating the MALAT1-miR-1914-3p-YAP axis to induce NSCLC drug resistance and metastasis. J Hematol Oncol (2019) 12(1):135. 10.1186/s13045-019-0830-6 31818312 PMC6902496

[21] Merrick D Kittelson J Winterhalder R Kotantoulas G Ingeberg S Keith R. Analysis of c-ErbB1/epidermal growth factor receptor and c-ErbB2/HER-2 expression in bronchial dysplasia: evaluation of potential targets for chemoprevention of lung cancer. Clin Cancer Res (2006) 12:2281–8. 10.1158/1078-0432.CCR-05-2291 16609045

[22] Yu H Paz-Ares L Yang J Lee K Garrido P Park K. Phase 1 Study of the Efficacy and Safety of Ramucirumab in Combination with Osimertinib in Advanced T790M-Positive EGFR-Mutant Non-Small Cell Lung Cancer. Clin Cancer Res (2020) 27:992–1002. 10.1158/1078-0432.CCR-20-1690 33046516 PMC8793125

[23] Zeng Z Li Y Pan Y Lan X Song F Sun J. Cancer-derived exosomal miR-25-3p promotes pre-metastatic niche formation by inducing vascular permeability and angiogenesis. Nat Commun (2018) 9(1):5395. 10.1038/s41467-018-07810-w 30568162 PMC6300604

[24] Lin S Negulescu A Bulusu S Gibert B Delcros J Ducarouge B. Non-canonical NOTCH3 signalling limits tumour angiogenesis. Nat Commun (2017) 8:16074. 10.1038/ncomms16074 28719575 PMC5520050

[25] Yao J Feng J Gao X Wei D Kang T Zhu Q. Neovasculature and circulating tumor cells dual-targeting nanoparticles for the treatment of the highly-invasive breast cancer. Biomaterials (2017) 113:1–17. 10.1016/j.biomaterials.2016.10.033 27794222

[26] Zhuo H Zhao Y Cheng X Xu M Wang L Lin L. Tumor endothelial cell-derived cadherin-2 promotes angiogenesis and has prognostic significance for lung adenocarcinoma. Mol cancer (2019) 18(1):34. 10.1186/s12943-019-0987-1 30832661 PMC6399986

[27] Korde A Jin L Zhang J Ramaswamy A Hu B Kolahian S. Lung Endothelial MicroRNA-1 Regulates Tumor Growth and Angiogenesis. Am J Respir Crit Care Med (2017) 196(11):1443–55. 10.1164/rccm.201610-2157OC PMC5736970 28853613

[28] Nie L Wu H Hsu J Chang S Labaff A Li C. Long non-coding RNAs: versatile master regulators of gene expression and crucial players in cancer. Am J Transl Res (2012) 4(2):127–50. PMC3353529 22611467

[29] Qi G Kong W Mou X Wang S. A new method for excavating feature lncRNA in lung adenocarcinoma based on pathway crosstalk analysis. J Cell Biochem (2019) 120(6):9034–46. 10.1002/jcb.28177 30582215

[30] Wang D Hu Y. Long Non-coding RNA PVT1 Competitively Binds MicroRNA-424-5p to Regulate CARM1 in Radiosensitivity of Non-Small-Cell Lung Cancer. Mol Ther Nucleic Acids (2019) 16:130–40. 10.1016/j.omtn.2018.12.006 PMC6411630 30861415

[31] Zhang X Chi Q Zhao Z. SPRY4-IT1Up-regulation of long non-coding RNA promotes tumor cell migration and invasion in lung adenocarcinoma. Oncotarget (2017) 8(31):51058–65. 10.18632/oncotarget.16918 PMC5584230 28881629

[32] Wu N Jiang M Liu H Chu Y Wang D Cao J. LINC00941 promotes CRC metastasis through preventing SMAD4 protein degradation and activating the TGF-β/SMAD2/3 signaling pathway. Cell Death Differ (2020) 28:219–32. 10.1038/s41418-020-0596-y PMC7853066 32737443

[33] Lu Q Shan S Li Y Zhu D Jin W Ren T. Long noncoding RNA SNHG1 promotes non-small cell lung cancer progression by up-regulating MTDH via sponging miR-145-5p. FASEB J (2018) 32(7):3957–67. 10.1096/fj.201701237RR 29466052

[34] Barbagallo D Caponnetto A Brex D Mirabella F Barbagallo C Lauretta G. CircSMARCA5 Regulates VEGFA mRNA Splicing and Angiogenesis in Glioblastoma Multiforme Through the Binding of SRSF1. Cancers (2019) 11(2):194. 10.3390/cancers11020194 PMC6406760 30736462

[35] Varricchi G Loffredo S Galdiero M Marone G Cristinziano L Granata F. Innate effector cells in angiogenesis and lymphangiogenesis. Curr Opin Immunol (2018) 53:152–60. 10.1016/j.coi.2018.05.002 29778674

[36] Cenni E Perut F Zuntini M Granchi D Amato I Avnet S. Inhibition of angiogenic activity of renal carcinoma by an antisense oligonucleotide targeting fibroblast growth factor-2. Anticancer Res (2005) 25:1109–13. 15868953

[37] Martins S Garcia E Luz M Pardal F Rodrigues M Filho A. Clinicopathological correlation and prognostic significance of VEGF-A, VEGF-C, VEGFR-2 and VEGFR-3 expression in colorectal cancer. Cancer Genomics Proteomics (2013) 10(2):55–67. 23603341

